# Effect of COVID-19 Pandemic on Orthopedic Surgery in Three Centers from Romania

**DOI:** 10.3390/ijerph18042196

**Published:** 2021-02-23

**Authors:** Dinu Vermeşan, Adrian Todor, Diana Andrei, Marius Niculescu, Emanuela Tudorache, Horia Haragus

**Affiliations:** 1 Department of Orthopedics and Trauma, Victor Babes University of Medicine and Pharmacy, Eftimie Murgu Square No 2, 300041 Timisoara, Romania; dinu@vermesan.ro (D.V.); horia.haragus@umft.ro (H.H.); 2 Department of Orthopedics, Traumatology and Pediatric Orthopedics, Iuliu Hatieganu University of Medicine and Pharmacy, Victor Babes Str No 8, 400000 Cluj-Napoca, Romania; adi.todor@yahoo.com; 3 Department of Rehabilitation and Physical Medicine, Victor Babeș University of Medicine and Pharmacy, EftimieMurgu Square No 2, 300041 Timișoara, Romania; 4 Faculty of Medicine, TituMaiorescu University, Dambovnicului Str No 22, District 4, 040441 Bucharest, Romania; 5 Department of Pulmonology, Victor Babeș University of Medicine and Pharmacy, Eftimie Murgu Square No 2, 300041 Timișoara, Romania; tudorache_emanuela@yahoo.com

**Keywords:** coronavirus infections, delivery of health care, Romania, hospitals, orthopedic procedures, pandemics

## Abstract

The COVID-19 pandemic has put an enormous burden on healthcare systems. As a direct consequence, many elective procedures were cancelled and available resources were relocated to emergencies and COVID-19 patients. We aimed to analyze the impact on orthopedic surgery in Romania. We performed a retrospective analysis of orthopedics and trauma cases admitted over the first six months of 2019 and 2020 in three representative clinics. In total, there were 1900 patients: 1241 from Timisoara, 216 from Cluj-Napoca, and 443 from Bucharest. In April, activity for all cases in the regional trauma center dropped to 23.8% and stopped in the other two. No arthroscopies or elective joint replacements were performed in April. By June, hospital admissions resumed for trauma cases while arthroscopies and joint replacements still lagged behind.

## 1. Introduction

The COVID-19 (corona virus 2019) pandemic had an unprecedented impact on our lives. Starting at the beginning of 2020, the entire world was forced to quickly adjust to using personal protective equipment (PPE) and physical distancing [1]. As the disease continued to spread, the high number of cases put an enormous burden on the healthcare systems. As a direct consequence, many elective procedures were cancelled and all available resources were relocated to emergencies and COVID-19 patients [1,2,3].

The orthopedic specialty was one of the most seriously hit due to the large number of postponable elective procedures and major surgeries with high resource requirements. Worldwide, joint replacements in particular were stopped and orthopedic departments were converted toCOVID-19 facilities [1,2,3,4,5]. However, while this process followed similar guidelines, it had significant regional variability [1,2,3,4,5,6,7,8,9].

In the USA (United States of America), on 14 March 2020, the Surgeon General recommended cancelling all elective surgeries. To help contain potential ambiguities, professional societies such as the AAOS (American Academy of Orthopedic Surgeons) issued guidelines and protocols [3]. The initial focus was to postpone all surgeries that could be delayed one month without causing harm. Additional emphasis was put on exclusively accepting outpatient treatments or limiting as much as possible the length of stay in hospital. Orthopedic emergencies were defined as surgeries that should be performed immediately or within the first 24 h. The Orthopedic Trauma Association insisted that whenever possible, fracture fixation should be performed as outpatient surgery. Pathologies considered non deferable electives were septic arthritis, malignant tumor, risk of impeding pathological fractures, neurological symptoms, traumatic tendon injury, dislocations and aseptic loosening of total joint replacement, and loose bodies/joint locking [4]. Additionally, a distinct new group of patients emerged: COVID-19-positives who required emergent surgery. As the pandemic progressed and evolved, more and more data became available. The American College of Surgeons and the Centers for Disease Control and Prevention cautioned that orthopedic surgery should be rare for COVID-19-positive or high-risk patients [9].

In Romania, the first case of COVID-19 was confirmed at the end of February 2020. On March 11, the WHO (World Health Organization) declared the novel coronavirus a pandemic. In an effort to stop the spread of the virus, a state of emergency was declared on 16 March. To prepare for the treatment of a potential surge in the number of cases, following global trends, elective procedures were cancelled and many orthopedic departments were converted to COVID-19 facilities [1,2,3,4,5,6,7,8,9]. The first wave of the disease was kept under control and the lockdown ended on 14 May. Throughout the following months, efforts were made to safely resume elective procedures while still maintaining a safe environment for patients and medical personnel.

The main orthopedics and trauma providers in Romania are run by the state owned, national healthcare system. The majority are part of major emergency county hospitals, while some are organized as orthopedics and rehabilitation units. The private sector was under development and included low to middle sized facilities. Historically, the most important centers were located in the capital (Bucharest) and the major cities Timisoara, Cluj-Napoca, Iasi, Tirgu-Mures, and Craiova.

In this study, we aimed to analyze the impact of the COVID-19 pandemic on the orthopedic surgery in Romania.

## 2. Materials and Methods

We performed a retrospective analysis of orthopedics and trauma cases admitted over the first six months of 2019 and 2020 in three representative clinics. A level I, university affiliated regional trauma center from Timisoara, where the bed number of the orthopedic facility analyzed was reduced to a third during the state of emergency. A private hospital from Cluj-Napoca where activity stopped during April and a general hospital from Bucharest where activity stopped in mid-March did not resume during the study period.

Hospital admissions, main diagnosis, and surgical procedures were retrieved from the electronic patient records and classified according to ICD-10 coding (the 10th revision of the International Statistical Classification of Diseases and Related Health Problems, a medical classification list by the World Health Organization). In order to minimize errors, two authors reviewed all data. Based on medical judgement, inconsistencies found between patient demographics, hospitalization, diagnosis, and procedure were further investigated and corrected.

Hospital admissions were defined as all patients admitted to any of the three orthopedic and trauma clinics included in the study within the selected timeframe. Elective procedures were defined as any arthroscopy or arthroplasty, irrespective of anatomic location, medical indication or implant type, performed on patients admitted to the hospital. Trauma admissions were defined as all hospital admissions with an ICD-10 code from the categories beginning with the letter ‘S’ (Injuries, poisoning and certain other consequences of external causes related to single body regions). Upper limb trauma was defined as hospital admissions coded using ICD-10 ‘S’ categories for the upper limb. Hip fractures were defined as hospital admissions coded using ICD-10 ‘S’ categories for fractures of the femoral neck, trochanteric, and subtrochanteric regions. Lower limb trauma was defined as hospital admissions coded using ICD-10 ‘S’ categories for lower limbexcluding the hip fractures defined above.

Results are presented as descriptive statistics using Office Excel spreadsheets (Microsoft, USA). Statistical significance was tested using mixed model two-way analysis of variance (ANOVA) for a *p* value <0.05 and 95% confidence interval. Analysis was performed using GraphPad Prism 9 (GraphPad Software, Inc., San Diego, CA, USA).

## 3. Results

During the study period, there were a total of 1900 patients, 1195 in 2019 and 705 in 2020. A total of 1241 were from Timisoara, 216 from Cluj-Napoca, and 443 from Bucharest.

### 3.1. Hospital Admissions

For the first two months of 2020, the number of inpatients slightly increased in all three hospitals, from a total of 177 (January) and 184 (February) in 2019 to 182 and 199 in 2020, respectively. On average, hospital admissions dropped more than half in March. In April, activity for all cases in the regional trauma center dropped to 23.8% and stopped in the other two. It slowly resumed to 30.3 and 72.6% in May and June in the regional trauma center, respectively, and remained closed in the general hospital. In contrast, the private hospital rebounded quicker. After being closed in April, in May and June, it had 141.7 and 104.2% of the activity from the previous year (Table 1 and Table 2).

### 3.2. Elective Procedures

In all three centers combined, arthroscopies dropped to 34.5% in March 2020. No arthroscopies were performed in April and only the private hospital resumed arthroscopies in May. Overall, in June, there was only one third of the volume of the previous year (Table 3 and Table 4).

Joint replacements followed a similar trend. However, data from the private hospital was biased by the low number of patients and high fluctuations (range 1–11 per month). Even so, joint replacements were resumed quicker and with a higher volume compared to the regional trauma center (Table 5 and Table 6).

### 3.3. Trauma Admissions

In March and April 2020, upper limb trauma admissions decreased to 64.3 and 45.5% of those of the previous year, respectively. Hip fractures (fractures of the femoral neck, trochanteric, and subtrochanteric regions) decreased to 50 and 36% and the rest of the lower limb trauma to 40.9 and 52.4%, respectively. Combined, admissions for extremity trauma decreased by half in March and April 2020 compared to 2019. These returned to normal in June (Table 7 and Table 8).

## 4. Discussion

In the first month after the state of emergency was issued in mid-March 2020, hospital admissions were reduced to half of those of the previous year. In April, two of the three facilities analyzed in our study stopped all orthopedic activity. In the regional trauma center, admissions were reduced to one third compared to the previous year. By June, the regional trauma center and the private hospital returned to normal, while the general hospital still remained closed in support for COVID-19 patients. Orthopedic surgery was able to resume quicker in the private clinic, probably due to the smaller scale and lack of COVID-19 support conversions.

The policy of general ‘’lockdown’ was adopted by most governments (including Romania) worldwide to prevent a rapid escalation in the number of cases in the first few weeks of the pandemic. By drastically reducing all activities and confining people to their houses, the interpersonal contact was significantly reduced. This kept the absolute number of infected cases low and thus medical systems gained sufficient time to get organized and prepared. Especially problematic was the shortage of PPE such as N95/PPE2 grade face masks and ICU (intensive care units) facilities to accommodate the most severe cases.

As the ‘lockdown’ measure was gradually instituted throughout the European Union, many people working abroad returned to Romania from the countries most severely hit by the pandemic such as Italy, Spain, France, and Germany, which led to additional difficulties in managing high volumes of people who required quarantine. In 2020, when the COVID-19 pandemic hit, Romania was a low to middle income country, located at the eastern part of the European Union. It had approximately 20 million inhabitants and medical service providers were mainly organized in state run major hospitals. The private sector was under development and included low to middle sized facilities.

In the USA (United States of America), on 14 March, the Surgeon General recommended cancelling all elective surgeries. To help contain potential ambiguities, professional societies such as the AAOS (American Academy of Orthopedic Surgeons) issued guidelines and protocols [3]. The initial focus was to postpone all surgeries that could be delayed one month without causing harm. Additional emphasis was put on exclusively accepting outpatient treatments or limiting as much as possible the length of stay in hospital. Orthopedic emergencies were defined as surgeries that should be performed immediately or within the first 24 h. The Orthopedic Trauma Association insisted that whenever possible, fracture fixation should be performed as outpatient surgery. Pathologies considered non deferable electives were septic arthritis, malignant tumor, risk of impeding pathological fractures, neurological symptoms, traumatic tendon injury, dislocations and aseptic loosening of total joint replacement, and loose bodies/joint locking [4]. Additionally, a distinct new group of patients emerged: COVID-19-positives who required emergent surgery. As the pandemic progressed and evolved, more and more data became available. The American College of Surgeons and the Centers for Disease Control and Prevention cautioned orthopedic surgery should be rare for COVID-19-positive or high-risk patients [9].

Nevertheless, in many circumstances, the lack of precision in stratifying activity led to unforeseen challenges [1,2,3,4,5,6,7,8,9]. Having to abruptly adopt a paradigm shift and decide what defined an emergency and who required mandatory surgical treatment meant many cases were treated conservatively or postponed. Ultimately the responsibility of the treating physicians, many fractures went on to develop mal- or non-union and joint stiffness.

Arthroscopic procedures and elective joint replacements were also drastically reduced during the ‘lockdown’ period in Austria, Germany, Greece, and Switzerland, while trauma cases reduced to up to one third of the pre-pandemic levels [2,3,4,5,6,7,8,9]. Turgut et al. reported that in Turkey, fractures decreased by approximately one third during the ‘lockdown’ period compared with previous two years, most likely due to less mobility on the streets. At the same time, the pediatric patients were younger, probably due to school closures and reduced access to playgrounds, which resulted in decreased adolescent fracture rates [7,8]. In our study, trauma admissions were only analyzed as subsets (upper limb, hip fractures, and the rest of the lower limb) in the regional trauma center due to the high number of cases compared to the other two. In May and June 2020, the increase in upper limb trauma was more prominent than for hip fractures and lower limb trauma. This may be a rebound effect of failed conservative treatments begun during the state of emergency.

As the first wave of the pandemic passed, additional facilities and supplemental resources became available and orthopedic departments gradually resumed elective procedures. However, with the significant increase in the number of infections in the population, virtually any patient who presented to the emergency rooms of acute trauma centers could be an asymptomatic carrier. In order to prevent in hospital dissemination of the SARS-CoV-2 (severe acute respiratory syndrome—coronavirus—2), healthcare facilities adopted different and sometimes divergent strategies. Where available, all patients were considered infected until proven otherwise. An emergency lung CT (computed tomography) was evaluated for typical COVID-19 lesions. Swabs were then taken for the RT-PCR (reverse transcription polymerase chain reaction) test, which sometimes required up to 24 h for the result to be available. Until then, the patients were given the same treatment as before the pandemic, at the expense of protective equipment consumption, designated circuits, separate rooms, and operating theaters. Another option was to provide only immediate care, put them into isolation, and proceed with definitive treatment using the pre-pandemic PPE standards, once the SARS-CoV2 PCR test results were negative [1,2,3,4,5,6,7,8,9].

Asymptomatic patients present with potential risk of respiratory failure, based on lung involvement as well as transmitting the disease to the operating team. Symptomatic patients are already very frail and additional stress may precipitate organ failure and death. They should be monitored perioperatively by a multidisciplinary team. If surgery cannot be postponed, acute orthopedic trauma centers should consider dedicated surgical teams, negative-pressure operating room, regional anesthesia, intubation and extubating outside of operating room, cardiopulmonary monitoring, use of advanced PPE, caution with high-speed power tools, and electrocautery during surgery [9,10].

Resuming elective surgeries has also been proven to be effective. It is important to understand that practices and protocols should be modified or changed in order to minimize the risk of viral transfer. Each hospital and health care facility should consider their unique situation in terms of SARS-CoV-2 presence, staffing, and PPE availability when determining how and when to implement these changes. All elective patients should be screened for SARS-CoV-2 by means of a thorough history, physical examination, and RT-PCR testing within three to seven days before elective surgery. Positive (COVID-19) patients should not undergo elective surgery. Elective surgery may be resumed if lockdown in the region has been terminated, the number of COVID-19 cases in the region has been declining, the hospital has the capacity to accept non-COVID-19 patients, the facility has an adequate supply of PPE, testing kits for the SARS-CoV-2 virus, and is able to perform surgery safely with low risk of viral transmission [9,10]. Both patients and providers should be educated on hand hygiene, wearing a mask and social distancing. Waiting rooms should be avoided, high contact surfaces cleaned regularly, visitors should have restricted access, and be updated by phone. Patients should be housed in single rooms [9,10]. Patient preference and anxiety were not directly analyzed in our study. Nevertheless, it was the author’s opinion and experience from clinical practice during the pandemic year that elective patients particularly exhibited a tendency to avoid health care facilities that were also busy COVID-19 support centers. In addition, they were prone to postpone treatments, which may require prolonged hospitalization.

The overall disruptive effect of the COVID-19 pandemic has had financial implications, even for the health care sector. The economic concerns have been compared to previous events and also the 2008–2009 recession. Up until now, the effects have been hard to forecast. During the last financial crisis from only a decade ago, the global banking and crediting has had an indirect delayed repercussion on patient access to funding for elective surgeries. However, this effect has not been very profound and is especially visible in countries without universal access to healthcare resources such as the USA, and less apparent in the European Union [11,12,13,14].

The orthopedic scientific output was one of the least affected. The drawbacks from the clinical settings probably presented an opportunity for reflection and analysis. All large scale scientific congresses, conferences, prestigious society annual meetings, hands-on trainings, and workshops were either canceled, postponed, or held online. On the other hand, education has suffered greatly from social distancing [15,16,17]. The switch to teaching students exclusively through virtual platforms and online sessions and reducing clinical patient volume, both medical students and orthopedics and trauma residents were deprived of bed side experiences and direct teacher–student transfer of clinical skills.

Vaccination against COVID-19 has started and the hopes are high. For the first time after nearly one year on the battlefield, predictions are positive and the future seems brighter. Nevertheless, the war is not over yet. The lessons learned should help us manage resources more efficiently [1,2,3,4,5,6,7,8,9,10,11,12,13,14,15,16,17]. Stratification of orthopedic surgery as either elective, urgent, or emergent can maintain a satisfactory level of services for short periods [1,2,3,4,8]. However, the cancelled electives will need to be addressed at a later date or maybe in smaller, ambulatory surgical centers. The shift has now changed from postponement to being able to provide full services while maintaining a safe work environment [3,10,11,12,13,14,15,16,17,18,19,20,21]. To cope with the expected larger volume of COVID-19 patients, some orthopedic facilities will continue to provide beds in support for the pandemic. A way to address shortages of medical staff and inpatient accommodation is to decrease the length of stay. Therefore, ambulatory surgery for elective joint replacements has been increasing in popularity [3,10,11,12,13,14,15,16,17,18,19,20,21].

The main limitation of our study is the retrospective design. Elective procedures included arthroscopy performed for acute trauma such as incarcerated ’bucket handle’ meniscus tear or arthroplasty performed for fractures such as those of the femoral neck in the elderly. In addition, the three hospitals included in the evaluation were very different in size, administration, and addressability, which decreased the value of the combined results. However, they are representative for the three main orthopedic surgery facilities in our country.

## 5. Conclusions

The COVID-19 pandemic has had a profound disruptive effect on orthopedic surgery in Romania. In April, orthopedic activity stopped entirely in two centers and was reduced to urgent cases in the third. By June, hospital admissions resumed for trauma cases while arthroscopies and joint replacements still lagged behind.

## Figures and Tables

**Table 1:** Hospital admissions/month 2020/2019 (*n*).

Location	Jan.	Feb.	Mar.	Apr.	May	Jun.	*p*
TM	131	117	67	29	44	85	
	115	117	152	122	145	117	0.063
CJ	22	25	14	0	17	25	
	18	12	25	22	12	24	0.759
Buc	47	57	23	1	0	1	
	44	55	58	54	57	46	0.037
All	182	199	104	30	61	111	
	177	184	235	198	218	187	0.048

TM = Timisoara; CJ = Cluj-Napoca; Buc = Bucharest; All = All three hospitals analyzed; *p* = 2-way mixed model ANOVA.

**Table 2:** Hospital admissions/month 2020/2019 (%).

Location	Jan.	Feb.	Mar.	Apr.	May	Jun.
TM	113.9	100	44	23.8	30.3	72.6
CJ	122	108.7	56	0	141.7	104.2
Buc	106.8	103.6	39.6	1.8	0	2.1
All	102.8	108.1	44.2	15.1	28	59.3

TM = Timisoara; CJ = Cluj-Napoca; Buc = Bucharest; All = All three hospitals analyzed.

**Table 3:** Arthroscopic procedures/month 2020/2019 (*n*).

Location	Jan.	Feb.	Mar.	Apr.	May	Jun.	*p*
TM	16	22	6	0	0	5	
	20	18	32	19	28	22	0.033
CJ	14	12	9	0	10	9	
	10	10	19	16	8	8	0.433
Buc	8	10	5	0	0	0	
	8	7	7	12	10	12	0.097
All	38	44	20	0	10	14	
	38	35	58	47	46	42	0.052

TM = Timisoara; CJ = Cluj-Napoca; Buc = Bucharest; All = All three hospitals analyzed; *p* = 2-way mixed model ANOVA. Includes all indications for knee, shoulder, ankle and elbow.

**Table 4:** Arthroscopic procedures/month 2020/2019 (%).

Location	Jan.	Feb.	Mar.	Apr.	May	Jun.
TM	80	122.2	18.7	0	0	22.7
CJ	140	120	47.4	0	12.5	11.2
Buc	100	142.8	71.4	0	0	0
All	100	125.7	34.5	0	21.7	33.3

TM = Timisoara; CJ = Cluj-Napoca; Buc = Bucharest; All = All three hospitals analyzed. Includes all indications for knee, shoulder, ankle, and elbow.

**Table 5:** Joint replacement procedures/month 2020/2019 (*n*).

Location	Jan.	Feb.	Mar.	Apr.	May	Jun.	*p*
TM	16	23	9	2	1	14	
	17	26	29	16	20	18	0.032
CJ	1	10	3	0	4	11	
	2	1	3	3	2	10	0.465
Buc	21	25	10	0	0	0	
	21	21	27	20	26	14	0.052
All	38	58	22	2	5	25	
	40	48	59	39	48	42	0.063

TM = Timisoara; CJ = Cluj-Napoca; Buc = Bucharest; All = All three hospitals analyzed; *p* = 2-way mixed model ANOVA. Includes all indications for hip (hemi, total, and revision arthroplasty), knee (unicompartmental, total, and revision arthroplasty), shoulder (hemi, reverse, and anatomic arthroplasty) and radial head replacement for fracture.

**Table 6:** Joint replacement procedures/month 2020/2019 (%).

Location	Jan.	Feb.	Mar.	Apr.	May	Jun.
TM	94.1	88.4	31	12.5	5	77.7
CJ	50	1000	100	0	200	110
Buc	100	119	37	0	0	0
All	95	120.8	37.3	5.1	10.4	59.5

Timisoara; Cluj-Napoca; Bucharest; All three hospitals analyzed; Includes all indications for hip (hemi, total, and revision arthroplasty), knee (unicompartmental, total, and revision arthroplasty), shoulder (hemi, reverse, and anatomic arthroplasty) and radial head replacement for fracture.

**Table 7:** Trauma admissions/month in the regional center from Timisoara 2020/2019 (n).

Location	Jan.	Feb.	Mar.	Apr.	May	Jun.	*p*
Upper limb	10	6	9	5	7	15	
	10	9	14	11	9	12	0.169
Hip fractures	17	16	8	9	12	21	
	13	14	16	25	23	22	0.181
Lower limb	30	16	9	11	16	16	
	31	13	22	21	15	15	0.295
All	57	38	26	25	35	52	
	54	36	52	57	47	49	0.166

Hip fractures: fractures of the femoral neck, trochanteric and subtrochanteric regions; Lower limb: excluding hip fractures; All three anatomic regions analyzed. *p* = 2-way mixed model ANOVA.

**Table 8:** Trauma admissions/month in the regional center from Timisoara 2020/2019 (%).

Location	Jan.	Feb.	Mar.	Apr.	May	Jun.
Upper limb	100	66.7	64.3	45.5	77.8	125
Hip fractures	130.8	114.3	50	36	52.2	95.5
Lower limb	96.8	123	40.9	52.4	52.1	95.5
All	105.5	105.5	50	43.8	74.5	106.1

Hip fractures: fractures of the femoral neck, trochanteric, and subtrochanteric regions; Lower limb: excluding hip fractures; All three anatomic regions analyzed.
